# Pharmacologic inhibition of MIF nuclease: A new treatment paradigm to treat cell death

**DOI:** 10.1002/ctm2.1044

**Published:** 2022-09-20

**Authors:** Devanik Biswas, Valina L. Dawson, Ted M. Dawson

**Affiliations:** ^1^ Neuroregeneration and Stem Cell Programs, Institute for Cell Engineering Johns Hopkins University School of Medicine Baltimore Maryland USA; ^2^ Department of Neurology Johns Hopkins University School of Medicine Baltimore Maryland USA; ^3^ Solomon H. Snyder Department of Neuroscience Johns Hopkins University School of Medicine Baltimore Maryland USA; ^4^ Department of Physiology Johns Hopkins University School of Medicine Baltimore Maryland USA; ^5^ Department of Pharmacology and Molecular Sciences Johns Hopkins University School of Medicine Baltimore Maryland USA

## 1 DNA DAMAGE: A UNIFYING THEME IN NERVOUS SYSTEM DISORDERS

Neurodegenerative diseases and disorders are characterized by the loss of neurons via numerous mechanisms, resulting in observable phenotypes, such as memory impairment, motor dysfunction and more. Discoveries in neurodegeneration research are driving forward biomarker and therapeutic development. However, at this time, there are no curative or disease‐slowing treatments for neurological disorders, including Alzheimer's disease, Parkinson's disease (PD), amyotrophic lateral sclerosis and Huntington's disease, and other neurodegenerative diseases. This is likely due to an incomplete understanding of each disease's unique, as well as, shared pathophysiology. Markers of DNA damage are common among these disorders and defective DNA repair likely contributes to neurodegeneration.^1^ The nuclear enzyme, poly‐ADP ribose (PAR) polymerase‐1 (PARP‐1), plays an important role in detecting and facilitating the repair of DNA damage.^2^ However, when PARP‐1 is overactivated, the product of its catalytic activity, PAR, initiates a regulated cell death program, designated parthanatos, that plays an integral role in many diseases.^3^ Analyzing PARP‐1 activity and its downstream signalling cascades may lead to fruitful clinical applications. Along these lines, herein, we discuss a new treatment paradigm targeting the final step of the parthanatos.

## 2 PARTHANATOS: A MAJOR CELL DEATH MECHANISM

Parthanatos is a regulated cell death mechanism driven by the hyperactivation of PARP‐1, often caused by hypoxic or oxidative stresses, or inflammation.^4^ It plays important role in ischemic or traumatic tissue injury in a variety of organs, diabetes, multiple sclerosis and several neurodegenerative diseases.^3^ The product of PARP‐1 activation, PAR, then acts as a scaffold for DNA repair enzymes where it facilitates the assembly of proteins involved in DNA repair.^2^ However, when PARP‐1 is overactivated, PAR leaves the nucleus where it binds and inhibits mitochondrial hexokinase, leading to reductions in ATP and nicotinamide adenine dinucleotide (NAD^+^).^5^, ^6^ PAR also interacts with apoptosis‐inducing factor (AIF), a mitochondrial oxidoreductase that predominantly resides in the mitochondrial intermembrane space, but also resides at the outer mitochondrial membrane. AIF is released from the mitochondria after PAR binds to AIF at its PAR binding domain.^7^, ^8^ PAR‐induced AIF release from the mitochondria leads to AIF translocation into the nucleus, setting in motion the final stages of parthanatos, which is characterized by genomic DNA cleavage into fragments greater than 20 kb.

The discovery that AIF mediated PARP‐1‐dependent cell death led to a more complete picture of the molecular mechanisms underlying parthanatos.^9^ Yet, considering that AIF does not possess any nuclease activity, the identification of the Parthanatos‐Associated AIF Nuclease (PAAN) responsible for genomic DNA cleavage remained elusive. In 2016, using a two‐step screening strategy, we identified macrophage migration inhibitory factor (MIF) as the first PAAN.^10^

## 3 MIF: THE PARTHANATOS EXECUTIONER

**FIGURE 1:**
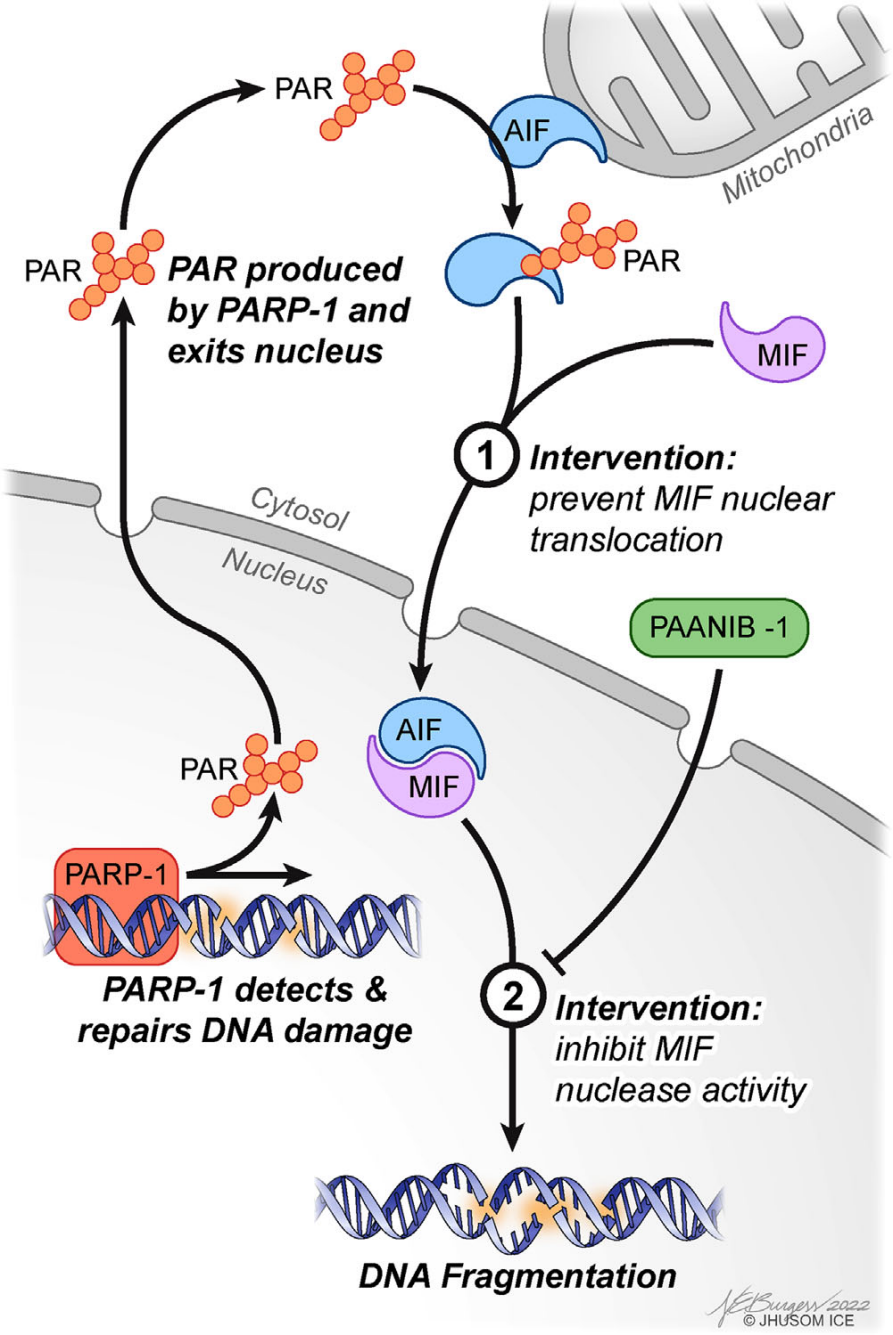
Macrophage migration inhibitory factor (MIF) involvement in cell death and potential intervention. Parthanatos begins with a stimulus that damages DNA, which results in poly‐ADP ribose (PAR) polymerase‐1 (PARP‐1) hyperactivation. As a byproduct of PARP‐1 catalytic facilitation of repair of DNA, the enzyme produces a polymer called PAR that can exit the nucleus. Once in the cytosol, PAR can bind to apoptosis‐inducing factor (AIF), promoting its translocation from the mitochondria. AIF then complexes with MIF, and together, they move to the nucleus, where MIF's gain of function nuclease activity cleaves genomic DNA, ultimately killing cells. Given that MIF is the final executioner of parthanatos, preventing MIF translocation to the nucleus or pharmacologically inhibiting its nuclease function with PAANIB‐1 may lead to new treatment paradigms for preventing cell death in several diseases where parthanatos plays a role.

MIF is a multifaceted protein with several functions, including its role as an atypical cytokine, tautomerase and oxidoreductase.^11^ MIF's nuclease activity is independent of its other functionalities.^10^ During parthanatos, MIF is carried into the nucleus by AIF where MIF's nuclease activity cleaves genomic DNA into 20‐ to 50‐kb fragments, concluding the final step of parthanatos (Figure 1).^10^ Disrupting MIF's interaction with AIF or its nuclease activity abolishes its ability to translocate into the nucleus and cleave DNA, respectively (Figure 1).^10^, ^12^ Interfering with MIF's nuclease activity, but not MIF's tautomerase activity, reduces neuronal injury and behavioural deficits after middle cerebral artery occlusion in mice.^10^ Moreover, MIF nuclease‐deficient (E22Q MIF) mice, but not MIF tautomerase‐deficient (P2G MIF) mice, are spared from PD pathology and the accompanying behavioural and motor deficits in a mouse model of sporadic PD.^13^ The fact that MIF's nuclease activity drives neuronal loss and is separable from its other functions prompted the exploration of specific inhibitors to MIF's nuclease activity while leaving MIF's other functions intact. Excitingly, our group discovered PAANIB‐1, a first‐in‐class brain‐penetrant inhibitor of MIF nuclease activity that does not interfere with MIF's tautomerase or cytokine activities.^13^ PAANIB‐1 provided robust neuroprotection and prevented neurobehavioural deficits in three mouse models of PD, highlighting its promise as a potential disease‐modifying therapy for disorders in which parthanatos plays a role.

## 4 FUTURE OUTLOOK

Our observations set the stage for the development of MIF nuclease inhibitors to treat a variety of diseases in which parthanatos plays a primary role. And since parthanatos drives pathology in many disorders, the impact of MIF nuclease inhibitors could be substantial.^3^, ^14^ Although parthanatos is characterized by PARP‐1 overactivation, clinically approved PARP‐1 inhibitors for the treatment of cancer have not been repurposed for other disorders.^15^ This is likely due to the fact that all the PARP‐1 inhibitors approved to date for the treatment of cancer act by trapping PARP‐1 on DNA thereby inhibiting homologous DNA repair.^16^ Impairment of DNA repair would not be desirable in disease of the nervous system^1^ and would likely have liabilities in other chronic disorders characterized by cell death. Thus, inhibiting parthanatos without affecting PARP activity is greatly desired. MIF nuclease inhibition may be the path to protect cells from injury induced by PARP‐1 overactivation. In particular, MIF nuclease activation is a gain of function activity that is set in motion by PARP‐1 overactivation. Inhibiting MIF's nuclease activity does not inhibit MIF's myriad of other functions, allowing MIF to carry out its other important functions while inhibiting its nuclease activity. Since MIF nuclease is not structurally similar to other human nucleases, MIF nuclease inhibitors would not interfere with the function of other human nucleases.^13^ Thus, to date, all indications suggest that it has minimal liabilities, while on the other hand, tremendous potential as a new treatment paradigm for cell death for a variety of disorders. Potent, selective and specific MIF nuclease brain‐penetrant inhibitors with appropriate safety and metabolic profiles hold particular promise for treating many neurodegenerative disorders^14^ and diseases beyond the nervous system.^3^ In summary, our findings and development of PAANIB‐1 will hopefully pave the way for future research involving MIF nuclease in several diseases and offer insights that can be readily translated to improving patient care in the clinic.

## 6 CONFLICT OF INTEREST

T.M.D. and V.L.D. are inventors of technology discussed in this publication, which Neuraly, Inc. is in the process of licensing from Johns Hopkins University. T.M.D. and V.L.D. are founders of Neuraly and hold shares of stock options as well as equity in Neuraly, Inc., which is a subsidiary of D&D Pharmatech Inc. This arrangement has been reviewed and approved by Johns Hopkins University in accordance with its conflict‐of‐interest policies.
